# Best practices for collecting repeated measures data using text messages

**DOI:** 10.1186/s12874-019-0891-9

**Published:** 2020-01-03

**Authors:** Noa’a Shimoni, Siripanth Nippita, Paula M. Castaño

**Affiliations:** 1 Department of Family Medicine, Rutgers New Jersey Medical School, 183 South Orange Avenue, E1562, Newark, NJ 07103 USA; 2 Department of Obstetrics and Gynecology, New York University-Langone Medical Center, New York, USA; 3 Obstetrics and Gynecology, Columbia University Irving Medical Center, New York, USA

**Keywords:** Electronic data capture, Mobile phone, Text messaging, Research methodology, Data collection

## Abstract

**Background:**

Researchers and clinicians use text messages to collect data with the advantage of real time capture when compared with standard data collection methods. This article reviews project setup and management for successfully collecting patient-reported data through text messages.

**Methods:**

We review our experience enrolling over 2600 participants in six clinical trials that used text messages to relay information or collect data. We also reviewed the literature on text messages used for repeated data collection. We classify recommendations according to common themes: the text message, the data submitted and the phone used.

**Results:**

We present lessons learned and discuss how to create text message content, select a data collection platform with practical features, manage the data thoughtfully and consistently, and work with patients, participants and their phones to protect privacy. Researchers and clinicians should design text messages to include short, simple prompts and answer choices. They should decide whether and when to send reminders if participants do not respond and set parameters regarding when and how often to contact patients for missing data. Data collection platforms send, receive, and store messages. They can validate responses and send error messages. Researchers should develop a protocol to append and correct data in order to improve consistency with data handling. At the time of enrollment, researchers should ensure that participants can receive and respond to messages. Researchers should address privacy concerns and plan for service interruptions by obtaining alternate participant contact information and providing participants with a backup data collection method.

**Conclusions:**

Careful planning and execution can reward clinicians and investigators with complete, timely and accurate data sets.

## Background

Clinicians and researchers sometimes need to collect repeated patient- and participant-reported data on a broad range of health outcomes. The information gathered relies on accurate self-reporting and is used to titrate medication, inform patient counseling, change practice patterns, and answer research questions. Traditionally, individuals record daily information using paper diaries and submit them weeks or months later. Information provided is oftentimes incomplete and diaries may be lost or damaged. Patients and participants may delay diary entry input until the last minute, which increases recall bias and decreases data validity. Using mobile phones to collect data may overcome these drawbacks [1–3].

People across different social and economic backgrounds regularly use mobile phones [4]. Using text messages to gather longitudinal or repeated measures data has unique advantages. Individuals are more likely to enter information in real time, which results in more reliable, complete data and minimizes recall bias [5–7].

The aim of this paper is to review project setup and management for successfully collecting patient-reported data through text messages.

## Methods

In this paper, we present lessons learned from our experience enrolling more than 2600 participants over 10 years in clinical trials that successfully used text messages to relay information or to collect data. For the purpose of this paper, we define text messages as a set of characters transmitted directly to a device without the use of instant messaging applications. We aggregate the studies and classify our recommendations into three areas: how to create the text message, manage the data, and work with mobile phones.

The studies that informed these recommendations include:

- Improving continuation in 962 oral contraceptive users via a randomized trial of daily text message reminders or standard care (Castaño, 2012) [8]
- Increasing rates of intra- and post-partum influenza vaccination in 1187 low-income pregnant women randomized to weekly text message reminders or standard care (Stockwell, 2014) [9]
- Collecting daily bleeding data in 230 participants who received an intrauterine device (IUD) and were randomized to either text messages or paper diaries (Nippita, 2015) [1]
- Demonstrating the feasibility of text messaging for influenza vaccine safety surveillance in 166 pregnant women (Stockwell, 2017) [10]
- Collecting daily bleeding data via text messages in 131 women for 90 days after IUD insertion early or late in the menstrual cycle (Shimoni, 2019) [11]

After aggregating the lessons learned from the listed studies, we conducted a narrative conceptual literature search to explore themes from other studies that have utilized repeated text messages to collect patient-reported data. We searched Ovid Medline for (“text messages” [mp] OR “Text Messaging” [MeSH]) AND (“data collection” [mp] OR “Data Collection” [MeSH]) from 1946 to August 12, 2019 without exploding the search to include the subheadings. We reviewed the full text or abstracts (when no full text was available) for all identified studies and included ones where participants responded directly to repeated text messages. We excluded studies where participants provided data only through an app or online survey. We assigned findings and recommendations from each qualifying study to the pre-determined headings of the text message, the data and the phone.

## Results

We obtained 204 results through a literature search and report on 29 studies relevant to this review. Table 1 details the key elements that researchers and clinicians may consider when using text messages for data collection: the message itself; the data and platform used to collect it; and the phone. The elements are borne of our experiences and recurred in the methodology and discussion sections of successful texting studies. Many are common sense study recommendations that are not likely to be formally evaluated but are useful prompts for investigators.

**Table 1:** A checklist of best practices for managing texts, data, and phones

The Text Message	
Create a short and simply worded text	
Ask the question and list the possible responses in the same text	
Discourage open ended responses when aggregated data is desired	
Identify the preferred time to text	
Share the exact text language with the participant at enrollment	
The Data	
Ensure the data platform is secure	
Minimize the transfer of sensitive information	
Validate responses, send error messages and recapture the data	
Send reminders when texts go unanswered	
Schedule staff to review the texted data regularly and contact participants to complete missing data	
Decide* a priori* how to manage missing data	
Prepare a data management protocol to append, correct, clean and order the data	
The Phone	
Enroll the phone, not just the participant	
Plan for disconnections or interruptions in service	
Discuss sensitive situations such as phone sharing	

### The text message

Text messages are well-suited for gathering time-sensitive information that may fluctuate, are well-received by participants, and yield response rates at least equivalent if not superior to paper, phone and internet. Participants from developed and developing countries respond at high rates to text message surveys. A 7-day study evaluating hunger hourly in 15 participants during waking hours yielded a 92% response rate within 30 min of the text prompt, with only two participants responding to fewer than 10 daily texts (out of 16) [12]. Another 7-day study where participants reported daily symptoms of irritable bowel syndrome by text message following a text prompt received 97% response within 10 h [13]. Studies of longer duration yield good response rates as well [10]. A study capturing vaginal bleeding and spotting for 90 days after IUD insertion achieved a 98% response rate by text [11].

Compared with other modalities for data collection, text messages often yield higher response rates than other modalities. Researchers studying adverse events following influenza vaccination among pregnant women reported a 90% response rate for participants using text messages, compared with 64% who received phone calls [10]. In a study of infant feeding practices, participant data from self-administered questionnaires or health visitors’ reports were more likely to have an unknown breastfeeding status compared with data collected from weekly text messages [6]. Participants in a study where investigators sent text messages twice a day to ask about post-operative pain and prescription opioid use achieved a 96% response rate, in comparison to the 66% that they previously achieved through internet-based surveys [7]. In Nippita et al’s study where participants were randomized to text versus paper diaries to submit daily bleeding data for 90 days, the median number of responses was 82 for text, compared with 36 for paper diaries [1]. Participants often prefer text messages for data reporting. Seven of the 102 participants who used text messages to submit diary data in the same study would have preferred paper diaries; in contrast, 47 of the 100 who used paper diaries would have preferred text messages [1]. In an observational study of influenza vaccine safety monitoring during pregnancy, 77% of participants preferred responding to questions via text message and only 5% would have preferred a paper diary [10].

Text messages are limited to 160 characters, which encourages investigators to ask only one question. Investigators with longer surveys may utilize a modular design where they divide surveys into successive questions that can be completed over short periods of time [14]. Researchers have successfully abbreviated surveys into a text format; one study used both modalities and found them to be equivalent [15].

**Fig. 1:**
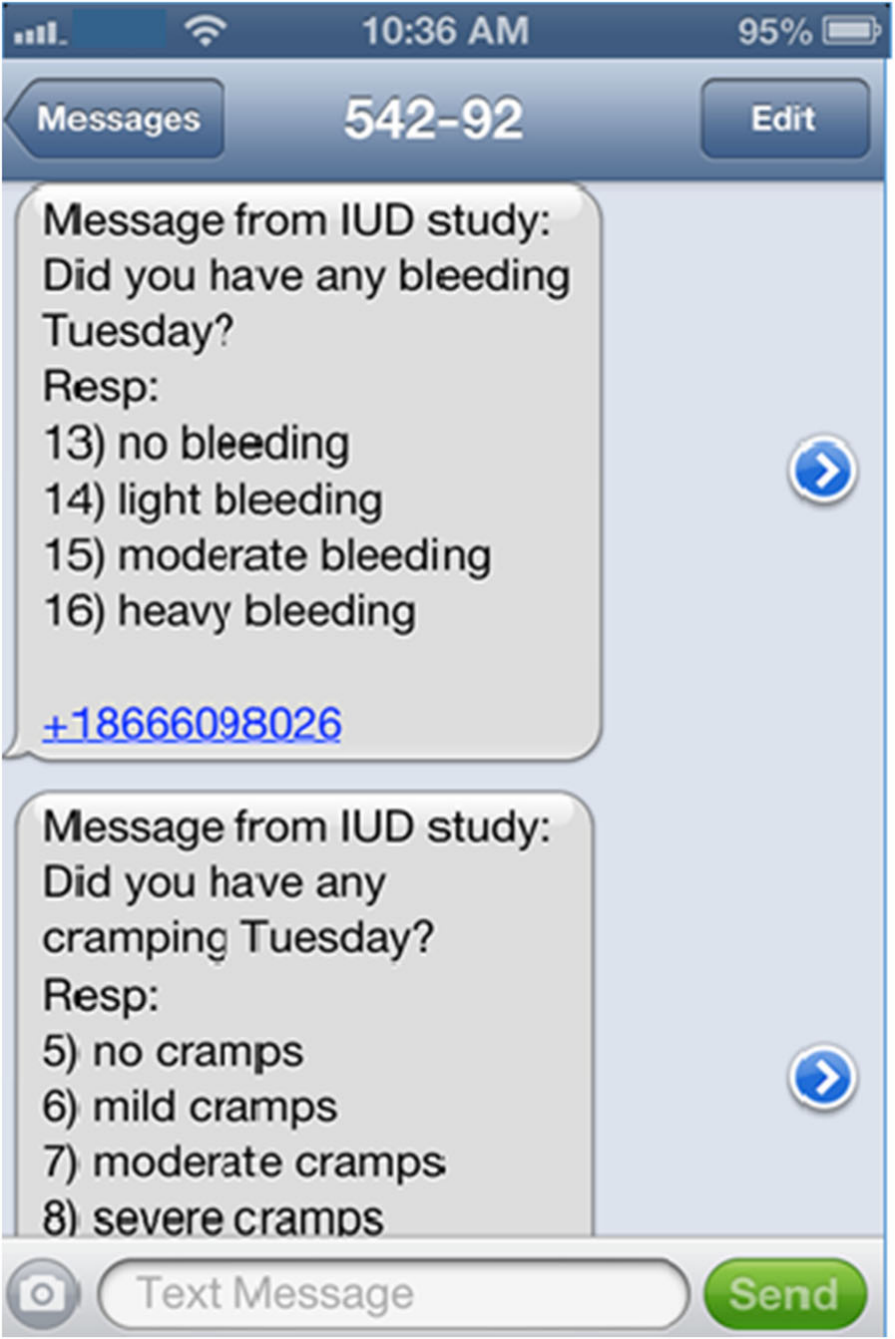
Text message including response options

Many investigators include the question and answer choices in the text prompt (Fig. 1) [1, 10, 11, 16, 17]. In cases where this is not feasible secondary to character restrictions, investigators often provide participants a copy of the question for reference [1, 11, 15]. Few of the studies we reviewed allowed text message responses to be open-ended. Investigators in Liberia during the 2014 Ebola emergency response used text messages to ask knowledge-based, cognitive, emotional, and social questions of individuals at risk for infection. They wanted to explore stigma with open-ended questions, but decided this was not feasible given the 160-character limitation and the additional training and post-coding that would be required of researchers [18].

Decisions need to be made regarding the optimal time to send text messages and may need to be individualized based on the variable collected. Brabyn et al. randomized participants with irritable bowel syndrome to morning or evening text collection. Those receiving evening text messages had a slightly higher response rate (94% versus 89%, *p* = 0.06) but there was no difference in time to response between the groups [13]. Investigators in a study that assessed low back pain in miners sent out messages each workday 15 min before the end of the shift and each weekend day at 4 pm and received similar response rates [19]. Turner et al. allowed participants to choose the time they would receive the text prompt each day. Other investigators also comment on participant preferences for text message timing as a factor that affects response rates [20, 21]. Since different variables need to be collected at different times, the specific time a message is sent may be less important than sending it at the same time each day.

Text messages may be an ideal modality for collecting data on stigmatized topics such as mental health, substance and alcohol use, or sexual behavior. In West et al’s study, participants were more likely to disclose substance use and mental health information when data were collected using text messages versus face to face interviews [14]. In Muwonge et al., couples preferred completing surveys on sexual activity and pre-exposure prophylaxis adherence via text message over in-person study visits (72% vs 28%) [17].

Participants in several studies found text messages to be encouraging and supportive, even when the text was a simple question [17]. Adolescents recovering from substance use disorders noted that text messages motivated them to maintain abstinence [22]. Similarly, in a study of men who have sex with men who reported their substance use after text messages, participants expressed that they liked having contact with someone who cared about their substance use [23].

### The data

Secure data platforms are advised for data collection [1, 10, 11, 19, 24]. Investigators in several studies were careful to remove as much identifying participant information as possible, using telephone numbers or participant IDs in the datasets [11, 25], recommending that participants immediately delete messages after answering them, and encouraging participants to password protect their phones [16, 17, 24].

**Fig. 2:**
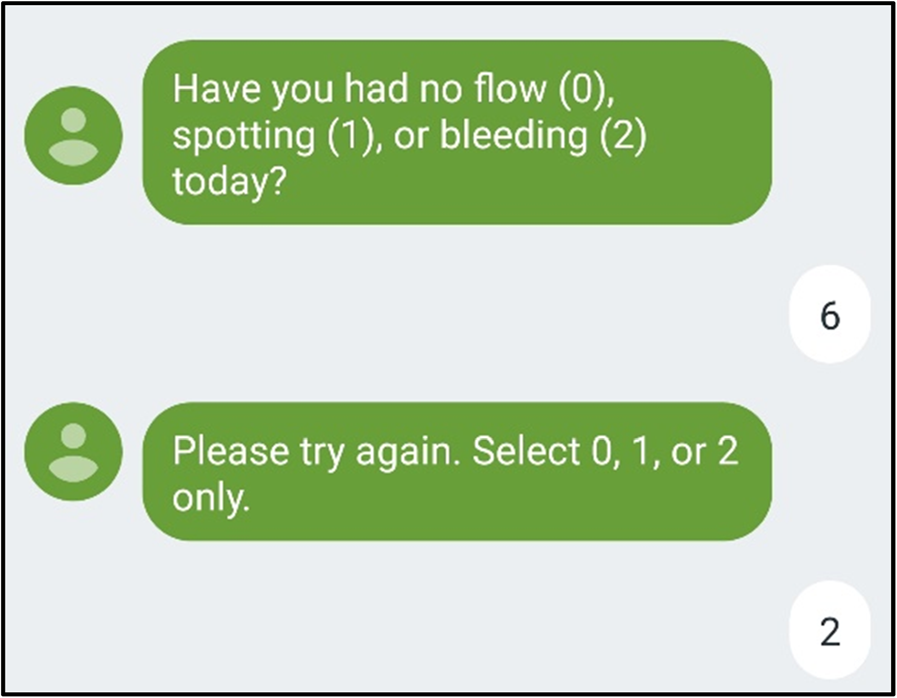
Real-time data validation automatically generates a follow-up text after an incorrect response

Investigators can take measures to validate data in real time. In Shimoni et al’s study, participants immediately received an error message if a response did not fall within the pre-specified options (Fig. 2).

Many studies use reminder messages to maximize response rates [1, 11, 26, 27]. Nippita et al. sent text messages daily at 9 pm and a reminder message 12 h later if the first message went unanswered [1]. Among the first 500 messages sent, 82% were answered after the first prompt, 6% after the second, and 12% were never answered. Shimoni et al. automatically sent a reminder text one hour after the initial text went unanswered. Participants responded to 84% of the initial prompts and to 14% of the reminder prompts; researchers obtained the remaining 2% of responses by contacting participants directly [11]. In both studies, the researchers limited the text messages to one reminder, reasoning that participants may experience text-fatigue following multiple reminders, which could compromise data collection.

Investigators may monitor incoming data on an ongoing basis, reaching out to participants by phone, email, or text within a few days of unanswered texts to achieve nearly complete data [11]. Stockwell et al. checked message reports daily and contacted patients with incomplete data. Response rates remained over 80% for participants still active 245 days into the study and only eight women never replied via text or phone [10]. Premkumar et al., who achieved a 96% response rate about pain and opioid use from post-operative participants, attribute their high response rates to a research assistant who followed up with participants after 3 days of no response [7].

Participants may call, email, or free-text messages in addition to their survey responses and research staff may reach out to participants to complete data. Researchers should have a preset plan in place for how to capture supplemental data. A study in Los Angeles utilizing a large electronic data collection system for HIV self-management and prevention/engagement of HIV-negative youth quickly realized that staff needed open-ended running notes to log ad-hoc interactions with participants [24].

Data monitoring may yield timely clinical information which may need to be acted on quickly, especially when investigators are collecting data on mental health symptoms. Researchers sending out a weekly text message containing a single item depression screen identified both individuals requiring medical attention with a psychotic episode and pranksters endorsing suicidal ideation during the course of the study [28].

### The phone

Participants occasionally fail to receive or respond to text message prompts. For Nippita et al., 33% of participants in the text message group either did not receive or could not respond to the initial prompts; technical difficulties could not be resolved for about half of this text message group and they switched to paper diaries. The remaining participants were subsequently able to text successfully. Lost or inactivated phones may also explain missed messages. For Castaño et al., 28% of 683 respondents reported service interruptions or changes during the 6-month follow-up period [8].

Use of pay-as-you-go phone plans can affect studies collecting data with text messages. Mobile providers of these plans frequently blocked messages that originated from “short” 5-digit numbers (as opposed to “long” 10-digit numbers). As a result, for Chernick et al. only 71% of the 1650 messages sent were successfully delivered [29]. In Gahleitner et al’s study collecting asthma and biometric data via text messages, even though a number of participants had pay-as-you-go phone plans, response rates were 82% or higher over 21 days of survey data collection [25].

Confidentiality concerns exist; if cell phones are shared, text messages may be seen by individuals who are not the intended recipients. For Castaño et al., phone sharing was rare, reported by only 2% of 968 participants [8]. In a study collecting sexual activity and pre-exposure prophylaxis adherence, 24% of participants said others saw their study messages but only 1 (3%) occurred without their permission and only 2 (6%) were bothered by this [17].

## Discussion

From our experience collecting repeated measures data, we learned valuable lessons and refined the process of collecting text message data. Our literature review was not intended to be a systematic review given the diverse nature of the data, but rather a synthesis of research experiences using text messages to collect patient-reported data. Exclusion of the narrower MeSH subheadings may have limited the number of articles reviewed. We created a set of recommendations, detailed as a checklist in Table 1. We primarily focus on research applications, but health care providers can apply these guidelines to clinical practice.

### The text message

Researchers may prompt participants to submit repeated measures data by texting a direct response, linking to a survey, or logging into another platform to enter the data [1, 5, 6]. Direct response eliminates a step and may yield better response rates.

Investigators must consider preferred language, reading comprehension level, and character limits when designing text message prompts [9, 18]. Standard English language text messages are limited to 160 characters; non-English characters may have lower limits (e.g., 67 for Chinese). Researchers can optimize response rates and data quality by keeping messages short and asking one question at a time [12–15]. Longer messages have downsides: they may be converted to multiple messages or truncated; may lose the participant’s attention; or may use up a limited text message plan, risking participant frustration and premature discontinuation. While text messaging may be used for open-ended responses, researchers should be mindful of character limits. A texted response longer than 160 characters may only be partially captured. Free-text responses also require more active data management. Additionally, investigators should ensure that text messages do not elicit personally identifiable information, which should not be exchanged with participants using this modality.

The participant should be introduced to the text message content along with the possible responses at the enrollment visit. Institutional review boards often require a protocol that details the text message wording, the time messages will be sent, and the frequency of texts. Instructions for how participants may discontinue text message prompts must be explicit; many platforms allow for a simple response to automatically enable this process. Clearly written guidelines help set consistent practices and facilitate data collection in both study and non-study settings.

### The data

The data platform sends out, receives, and stores text messages and must be secure. The cost and features of the data handling systems vary widely [9]. Vendors may charge an initial setup fee, a monthly access fee, and a per participant fee. Database vendors commonly offer pricing plans based on the number of messages their clients anticipate sending; investigators must thus determine this in advance. They may apply additional per-message charges if the number of text messages sent exceeds the number specified in the contract. Investigators may want to explore the messaging functionality of REDCap, a secure data collection platform used in academic research settings. Those who use this database to store data may send and receive texts through a collaborating text message provider. This may be more affordable, since the researcher creates and manages the database and the cost is based only on the number of text messages sent and received. REDCap surveys are flexible and researchers can allow participants to respond by text, web, or voice call.

Investigators must decide when to send texts and whether to send reminders. Some platforms may allow investigators to customize the text release time individually for each participant [8]. Depending on the type of data researchers collect, messages should be delivered at a consistent time during the day [13, 21, 30]. Many studies demonstrate that reminders increase the response rate [1, 11, 31]; however, data demonstrate that one reminder may be optimal and repeated alerts are often ignored [27]. Researchers and clinicians should consider the type of data they are collecting when deciding how frequently to send text messages and reminders.

When researchers regularly track participant responses, they rapidly identify missing replies, and quickly prompt participants to submit missing information. Close data monitoring is time- and resource-intensive, but can result in a nearly complete and accurate dataset collected in close-to real time.

Vendors may offer several features to facilitate data handling. Messages can be automated and customized. The vendor should provide time stamps for responses to facilitate data cleaning and interpretation. Investigators should inquire which data are captured (outgoing messages, incoming messages, or both), whether the vendor provides real-time access to raw data, and how long the vendor will allow access to the data.

Investigators should create *a priori *guidelines for how to append or correct data. If a participant responds multiple times to the same text prompt, which response should be selected by the investigator? It is impossible to anticipate all scenarios that may arise, but setting as many parameters as possible in advance of data cleanup improves consistency. Investigators should also define a protocol for pursuing missing data points based on available time and resources. Protocols should include whether, when, and how to contact participants regarding missing data and how to impute missing values. Researchers who maintain a file with all such communication for each participant are rewarded with the ability to refer to those notes and append missing data with greater confidence.

Text messages sent by phone are not encrypted during transmission. For this reason, we recommend limiting transmitted protected health information (PHI). Of note, phone numbers are considered PHI and transmission of this variable during a study should be reviewed with the participant during the consent process. Investigators who wish to encrypt all transmitted data have options beyond text messages. They may use apps that utilize SSL encryption (a security protocol that encrypts data in transit similar to online banking apps that could allow omission of PHI entirely by utilizing an assigned identification number) or may send the participant a link to a secure survey. If transmitting data by Wi-Fi, participants should ensure they are using a secure network.

Depending on the database used, messages may pass or be stored on external servers that are not protected by a firewall. Many academic institutions require that data containing PHI be stored behind a firewall.

When selecting a vendor or database, investigators should inquire if the data are stored in an encrypted environment that conforms to regulations such as the United States’ Health Insurance Portability and Accountability Act or the European Union’s General Data Protection Regulation. Participants should be informed during the consent process that the database vendor will have access to study data. When we use an external vendor to store data, our institutional review board and legal counsel vet the agreement to comply with health information privacy regulations.

### The phone

Participants need to have a working mobile phone to participate in studies that rely on text messages for data collection. The study’s inclusion criteria should state this explicitly. To ensure the participant has a working phone, investigators should send a trial text message during enrollment and confirm the response was captured in the database. This process verifies that the phone can receive messages and the participant can successfully respond to them. Despite these efforts, investigators should bear in mind that unforeseen technical difficulties may still occur. As technology changes, barriers to data collection change: new barriers arise and others diminish. We cannot always predict what the new barriers will be, but ongoing data monitoring can identify challenges early in a study and mitigate data loss.

Researchers should document alternate methods to contact participants and encourage participants to contact the study team for any cellular service changes. When disconnections or interruptions in service occur, offer the participant an alternate way to keep the data or share it with study staff, such as email, online submission, or, as a last resort, paper records.

While participants must explicitly consent to receive messages, there are cases where others may view potentially sensitive information. Researchers who address phone sharing concerns in advance of enrollment can raise participant awareness and help problem solve or prevent potentially uncomfortable situations that can adversely impact the participant or undermine data collection. A participant who cannot ensure they are the only users of a cell phone, especially when sensitive data will be requested, may not be the best candidate to report data via text messages. Suggestions for minimizing security breaches include providing minimal identifying information to the text message vendor, allowing participants to create an alias if messages will address the recipient by name [5], encouraging participants to password-protect access to their phone [5, 17], and recommending participants delete the text messages after responding [17].

## Conclusions

Researchers who gather information on repeated measures can collect a rich and more complete data set using text messages than by using standard data collection methods. We have outlined important points regarding the text message, the data, and the phone for investigators who are considering using this modality. We have highlighted sensitive issues so that potential problems can be mitigated with careful attention to the checklist. Our experience and expertise derives from reproductive health and preventive medicine but our review of the pertinent, related literature demonstrates these same principles apply broadly to patient-reported data collection in varied fields.

## Data Availability

The data used within this article are published and accessible through Pubmed. Raw data sets are not available.

## Abbreviations

HIV: Human Immunodeficiency Virus
ID: Identification
IUD: Intrauterine device
MeSH: Medical Subject Headings
mp: Refers to combined search fields in Ovid Medline
PHI: Protected Health Information
SSL: Secure Sockets Layer
